# CDKN2A loss-of-function predicts immunotherapy resistance in non-small cell lung cancer

**DOI:** 10.1038/s41598-021-99524-1

**Published:** 2021-10-08

**Authors:** Stanley I. Gutiontov, William Tyler Turchan, Liam F. Spurr, Sherin J. Rouhani, Carolina Soto Chervin, George Steinhardt, Angela M. Lager, Pankhuri Wanjari, Renuka Malik, Philip P. Connell, Steven J. Chmura, Aditya Juloori, Philip C. Hoffman, Mark K. Ferguson, Jessica S. Donington, Jyoti D. Patel, Everett E. Vokes, Ralph R. Weichselbaum, Christine M. Bestvina, Jeremy P. Segal, Sean P. Pitroda

**Affiliations:** 1 Department of Radiation and Cellular Oncology, The University of Chicago, 5758 S Maryland Ave, MC 9006, Chicago, IL 60637 USA; 2 Pritzker School of Medicine, The University of Chicago, Chicago, IL USA; 3 Section of Hematology/Oncology, Department of Medicine, The University of Chicago, Chicago, IL USA; 4 Department of Pathology, The University of Chicago, Chicago, IL USA; 5 Section of Thoracic Surgery, Department of Surgery, The University of Chicago, Chicago, IL USA; 6 Section of Hematology/Oncology, Department of Medicine, Northwestern University, Evanston, IL USA; 7 Ludwig Center for Metastasis Research, The University of Chicago, Chicago, IL USA

**Keywords:** Non-small-cell lung cancer, Immunotherapy, Cancer genomics

## Abstract

Immune checkpoint blockade (ICB) improves outcomes in non-small cell lung cancer (NSCLC) though most patients progress. There are limited data regarding molecular predictors of progression. In particular, there is controversy regarding the role of CDKN2A loss-of-function (LOF) in ICB resistance. We analyzed 139 consecutive patients with advanced NSCLC who underwent NGS prior to ICB initiation to explore the association of CDKN2A LOF with clinical outcomes. 73% were PD-L1 positive (≥ 1%). 48% exhibited high TMB (≥ 10 mutations/megabase). CDKN2A LOF was present in 26% of patients and was associated with inferior PFS (multivariate hazard ratio [MVA-HR] 1.66, 95% CI 1.02–2.63, *p* = 0.041) and OS (MVA-HR 2.08, 95% CI 1.21–3.49, *p* = 0.0087) when compared to wild-type (WT) patients. These findings held in patients with high TMB (median OS, LOF vs. WT 10.5 vs. 22.3 months; *p* = 0.069) and PD-L1 ≥ 50% (median OS, LOF vs. WT 11.1 vs. 24.2 months; *p* = 0.020), as well as in an independent dataset. CDKN2A LOF vs. WT tumors were twice as likely to experience disease progression following ICB (46% vs. 21%; *p* = 0.021). CDKN2A LOF negatively impacts clinical outcomes in advanced NSCLC treated with ICB, even in high PD-L1 and high TMB tumors. This novel finding should be prospectively validated and presents a potential therapeutic target.

## Introduction

Over the past two decades, major developments have been made in the molecular characterization and treatment of non-small cell lung cancer (NSCLC). Approximately 30% of NSCLCs harbor oncogenic driver mutations that can be targeted by a growing number of small molecule inhibitors^1–4^. Immune checkpoint blockade (ICB), with or without chemotherapy, has had a significant impact on disease outcome. In the metastatic setting, seminal KEYNOTE, CheckMate, and IMpower studies have demonstrated improvements in clinical outcomes over standard-of-care with the use of pembrolizumab +/− standard cytotoxic systemic therapy, nivolumab +/− ipilimumab, or atezolizumab + chemotherapy, respectively^5–10^. In the setting of unresectable locally advanced disease, the PACIFIC trial demonstrated improvements in progression-free (PFS) and overall survival (OS) with the addition of durvalumab following standard chemoradiation^11,12^. Numerous ongoing clinical trials are investigating the addition of ICB earlier in the course of disease management, including as neoadjuvant therapy for resectable disease (NCT02927301, NCT03158129).

Despite these practice-changing results, limited biomarkers are available to predict response rates and clinical outcomes following ICB. Validated biomarkers predictive of ICB response include high PD-L1 expression (≥ 50%) and high tumor mutational burden (TMB)^13,14^. Conversely, a subset of NSCLCs defined by KRAS/STK11 co-mutation has been found to be associated with a “cold” tumor immune microenvironment and marked resistance to anti-PD-1/PD-L1 therapy^15^. Importantly, these molecular features are absent in the majority of NSCLCs and when present have a limited correlation with response. Only one third of NSCLCs exhibit high PD-L1 expression^16^, less than one fifth exhibit KRAS/STK11 co-mutation^15^, and definitions of high TMB remain non-standardized^14,17^. In addition, even NSCLCs exhibiting both high PD-L1 and high TMB typically have ORRs of approximately 50%^13,14^.

Data has emerged more recently linking alterations in CDKN2A, an important tumor suppressor gene^18^, with ICB resistance in several solid tumors^19^. However, the largest of these studies found no association of CDKN2A loss-of-function (LOF) with ICB treatment outcome in NSCLC^20^. Given that CDKN2A silencing occurs in up to 40% of lung adenocarcinomas^3^ and is potentially targetable with clinically available CDK4/6 inhibitors^21^, there is a critical need to further characterize the relationship between CDKN2A LOF and treatment outcome following ICB. We therefore examined this association in a serial cohort of patients with NSCLC treated with ICB who underwent next-generation genomic sequencing (NGS) at our institution.

## Materials and methods

We investigated patients with locally advanced or metastatic NSCLC treated with ICB who had tumor NGS performed prior to the initiation of ICB. NGS was performed routinely for all patients as per institutional policy using either our institutional OncoPlus panel, a circulating tumor DNA (ctDNA) assay such as Guardant, or another third party tumor specimen-based panel. The OncoPlus panel is a validated 1,212-gene proprietary hybridization capture-based genomic sequencing assay as previously described^22^ (Supplementary Materials and Methods). In order to maximize the uniformity and depth of the genomic analysis while minimizing selection bias, only patients with OncoPlus performed on a tumor biopsy prior to ICB initiation were included; those undergoing NGS solely with an external commercial genomic sequencing assay were excluded. In total, 426 patients treated with ICB between 2016 and 2020 were screened for our study and 139 were eligible for analysis based on the above criteria. Copy number variations (CNVs) and tumor mutational burden (TMB) were quantified as described in the Supplementary Materials and Methods. Variants were interpreted based on the 2017 AMP, ASCO, and CAP standards and guidelines for the interpretation and reporting of sequence variants in cancer^23^. In particular, CDKN2A LOF was defined as pathogenic mutation or copy number loss. Demographic, clinical, pathologic, genomic, therapeutic, and radiographic data were retrospectively collected. The indications for ICB, specific ICB agents received, and the concomitant use of cytotoxic therapy were recorded. AJCC 8th edition clinical stage was recorded at initial cancer diagnosis and at the time of ICB initiation. A prior history of other malignancy was allowed if curative therapy had been rendered and the patient had no evidence of disease.

Per REMARK guidelines^24^, findings were externally validated using a previously reported, publicly available cohort of metastatic NSCLC treated with ICB at Memorial-Sloan Kettering Cancer Center (MSKCC)^25^. Demographic, clinical, therapeutic, genomic, and progression-free survival data for this cohort were downloaded from cbioportal (https://www.cbioportal.org/). Overall survival was not publicly available. Heavily pre-treated patients (> 5 previous lines of therapy) and those without available PD-L1 expression or TMB data within this cohort were excluded in order to ensure a comparable cohort for analysis. In order to confirm that any discovered effects were specific to the ICB setting, three previously reported, publicly available cohorts of NSCLC treated in the pre-immunotherapy era were also downloaded from cbioportal and queried^3,26,27^.

We examined PFS and OS from the date of ICB initiation as well as the ORR and the disease control rate (DCR). During ICB treatment, patients underwent clinical follow-up with standard surveillance imaging typically every 2–3 months. PFS was defined as the time from ICB initiation to disease progression or death. The sources for dates of death included the electronic medical record, the social security death index, and our institution’s cancer registry. ORR (partial response (PR) + complete response (CR)) and DCR (PR + CR + stable disease (SD)) were calculated using RECIST version 1.1 for those patients with evaluable lesions^28^. Irradiated lesions were excluded from these calculations unless there was radiographic disease progression. PFS and OS were analyzed using the Kaplan–Meier method with log-rank tests and Cox proportional hazards regression models. Covariates potentially prognostic for PFS and OS or predictive of ICB response were selected a priori and included in the *full* multivariate (MVA) model. Backward selection criteria were also used to select covariates with a *p* value < 0.1 on univariate analysis for inclusion in the *final* MVA model. Data were analyzed with JMP Version 14 for Windows software (SAS Institute, Cary, NC) and R (R Core Team, 2020).

All methods were carried out in accordance with a University of Chicago IRB-approved clinical database (IRB# 20-0284; PI: Sean Pitroda). The need for informed consent was waived by the University of Chicago IRB. The study was performed in accordance with the Declaration of Helsinki.

## Results

### Patient, tumor, and treatment characteristics

One hundred thirty-nine patients who received at least one cycle of ICB and met the pre-specified eligibility criteria were included in the analysis. The cohort was representative of the general population of patients with NSCLC (Table 1), with a median age of 66 (range, 35–91) and a smoking history in 88%. Most patients had adenocarcinoma (87%) and distant metastases at ICB initiation (89%). Of the tumors with PD-L1 status available (82%), 27% were PD-L1 0%, 32% were PD-L1 1–49%, and 41% were PD-L1 ≥ 50%. Approximately half of tumors (48%) had high TMB, defined as ≥ 10 mutations/megabase^14^; using a more stringent cut-off of ≥ 13.8 mutations/megabase, one third (32%) of tumors exhibited high TMB^17^.

**Table 1:** Patient and treatment characteristics.

	Entire cohort	CDKN2A LOF (N = 36)	CDKN2A WT (N = 103)	*p* value
**Age (mean, range)**	66 (35–91)	65 (42–91)	66 (35–89)	0.64*
**Sex**				0.79
Male	63 (45%)	17 (47%)	46 (45%)	
Female	76 (55%)	19 (53%)	57 (55%)	
**BMI (mean, range)**	25 (16–44)	26 (17–37)	25 (16–44)	0.48*
**Positive smoking history**	122 (88%)	31 (86%)	91 (88%)	0.73
**Histology**				0.0016
Adenocarcinoma	121 (87%)	26 (72%)	95 (92%)	
Squamous cell carcinoma	11 (8%)	4 (11%)	7 (7%)	
Other/Unknown	7 (5%)	6 (17%)	1 (1%)	
**Brain metastases prior to ICB**	43 (32%)	6 (18%)	37 (37%)	0.041
**ECOG 0–1**	119 (86%)	32 (89%)	87 (84%)	0.51
**TMB**				
Mean (range)	12 (0.7–88)	12.4 (1–49.5)	12.5 (0.7–88)	0.95*
≥ 10	66 (48%)	17 (47%)	49 (48%)	0.97
≥ 13.8	44 (32%)	10 (28%)	34 (33%)	0.56
**PD-L1 status (if available)**				0.13
0%	31 (22%)	13 (41%)	18 (22%)	
1–49%	36 (26%)	9 (28%)	27 (33%)	
≥ 50%	47 (34%)	10 (31%)	37 (45%)	
**Indication for ICB**				0.74
Stage IV treatment naïve	73 (53%)	17 (47%)	56 (54%)	
Stage IV recurrent/refractory	50 (36%)	14 (39%)	36 (35%)	
Stage III (neo)adjuvant	16 (11%)	5 (14%)	11 (11%)	
**ICB paradigm**				0.67
ICB alone	89 (64%)	22 (61%)	67 (65%)	
Combination therapy	50 (36%)	14 (39%)	36 (35%)	

*p* value determined using Chi-squared test unless otherwise noted.

*Two-tailed Student’s *t*-test.

All patients received one or more anti-PD-1/PD-L1 monoclonal antibodies during their ICB treatment course (58% pembrolizumab, 27% nivolumab, 12% atezolizumab, and 8% durvalumab) and 13% received concomitant anti-CTLA4 (ipilimumab) therapy. Most patients received ICB monotherapy (64%), with the remainder receiving chemo-immunotherapy, primarily with platinum-containing regimens (Supplemental Table 1). ICB was initiated in the setting of treatment-naïve metastatic disease (53%), treatment-refractory or recurrent metastatic disease (36%), or less commonly in the adjuvant/consolidative (standard-of-care) or neoadjuvant (clinical trial) setting for locally advanced, unresectable disease (11%). Median lines of systemic therapy prior to ICB in non-treatment-naïve patients was 1 (range 1–7) (Supplemental Table 1).

### Clinical outcomes on ICB

With a median follow-up of 11 months after ICB initiation (range, 0.2–44 months), median PFS was 6.5 months, 1-year PFS was 35%, and 2-year PFS was 21% in the entire cohort. The median OS was 15.9 months, 1-year OS was 60%, and 2-year OS was 41% in the entire cohort. In patients with locally advanced disease treated with definitive chemoradiation and ICB, 2-year PFS and OS were 44% and 57%, respectively, as compared with 18% and 39% in those who received ICB for metastatic disease.

Using Cox regression analysis, ICB indication (non-metastatic vs. metastatic, *p* = 0.055), female sex (*p* = 0.085), and TMB as a continuous variable (*p* = 0.080) were associated with trends towards improved PFS (Supplemental Table 2, columns 2 and 3). In patients with metastatic disease, increasing TMB as a continuous variable was associated with improved PFS (HR 0.97 per unit change, 95% CI 0.94–0.99, *p* = 0.016). PD-L1 ≥ 50% was not associated with PFS in the overall cohort or by ICB indication.

Using Cox regression analysis, increasing body mass index (BMI) (*p* = 0.024) and Eastern Cooperative Oncology Group performance status (ECOG) (0–1 vs. 2–3, *p* = 0.034) were associated with improved OS in the entire cohort (Supplemental Table 3, columns 2 and 3). In particular, patients with ECOG 0–1 had significantly improved OS as compared with those whose ECOG was 2–3 (median OS 17.0 months vs. 6.9 months, HR 0.49, 95% CI 0.28–0.95, *p* = 0.034). Patients with metastatic disease also had a trend towards worsened OS with increasing age at diagnosis (*p* = 0.094). Other variables, including PD-L1 status and TMB, were not associated with OS.

### Genomic landscape

**Figure 1:**
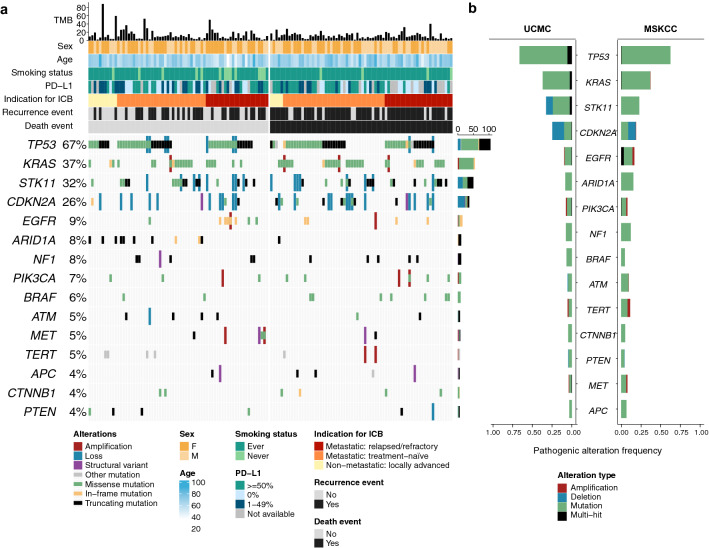
Genomic landscape of NSCLC tumors. (**a**) OncoPrint plot of UCMC cohort demonstrating top 15 recurrently altered genes. TMB, tumor mutational burden (mutations per megabase). PD-L1, Programmed death ligand-1. ICB, immune checkpoint blockade. OncoPrint plots were generated using the ComplexHeatmap package in R. (**b**) Frequencies of most commonly altered genes in UCMC and Memorial-Sloan Kettering Cancer Center (MSKCC) cohorts.

All patients underwent OncoPlus NGS testing of at least one lesion prior to the initiation of ICB. The most common somatic pathogenic genomic alterations were in TP53 (67%), KRAS (37%), STK11 (32%), CDKN2A (26%), and EGFR (9%). The associations of each pathogenic genomic alteration with other clinicopathologic features are depicted in Fig. 1a. A comparison of genomic alteration frequencies of the top altered genes between our cohort and the MSKCC cohort is presented in Fig. 1b and demonstrates a similar profile. The de-identified genomic data from our cohort is provided in Supplemental Table 4.

**Figure 2:**
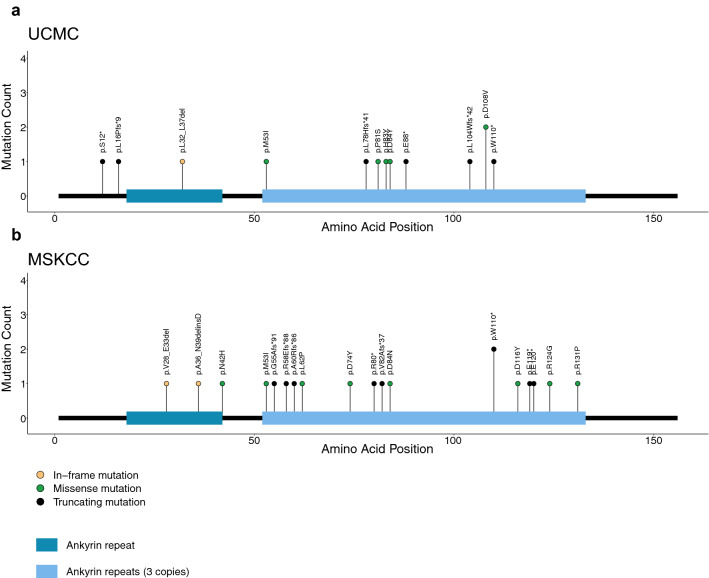
Genomic distribution of CDKN2A mutations. (**a**) UCMC cohort. (**b**) MSKCC cohort. Shown are amino acid changes due to mutations. Of note, loss-of-function due to gene loss are not presented in plot.

Of tumors exhibiting pathogenic CDKN2A variants, genomic loss/deletion occurred in 58%, whereas non-synonymous mutations occurred in 33%. The remaining tumors harbored structural variants (6%) or multiple hits (mutation and loss, 3%) of CDKN2A. The distribution of mutations in CDKN2A was similar to that found in the MSKCC cohort (Fig. 2a,b).

### CDKN2A loss-of-function and clinical outcomes

On univariate analysis, we identified a negative association between OS and CDKN2A LOF (HR 1.84, 95% CI 1.06–3.06, *p* = 0.05). We therefore compared other clinicopathologic and genomic features between patients with CDKN2A LOF tumors and CDKN2A wild-type tumors and found that the groups were well-balanced; in particular, there were no significant differences between groups with regards to variables predictive of ICB-associated outcomes (Table 1). In addition, there were no differences in the incidence of CDKN2A LOF alterations between PD-L1 high versus low tumors (21% vs. 33%, Chi-squared *p* = 0.17) or TMB high versus low tumors (TMB cutoff: 10 mutations/megabase: 26% vs. 26%, *p* = 0.97; 13.8 mutations/megabase: 23% vs. 27%, *p* = 0.56).

**Figure 3:**
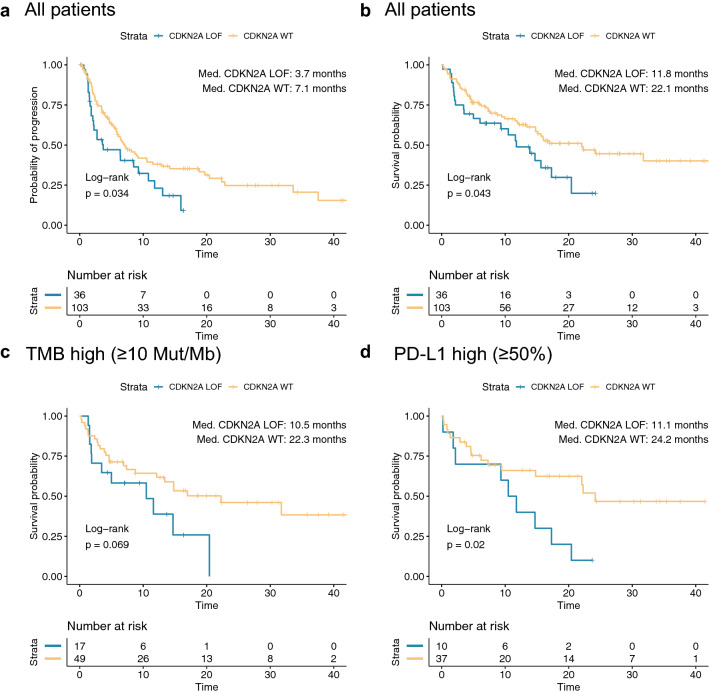
CDKN2A LOF and survival. Kaplan–Meier curves of patients with CDKN2A wild-type (WT) and loss-of-function (LOF) NSCLC demonstrating (**a**) PFS for entire cohort, (**b**) OS for entire cohort, (**c**) OS for high TMB subset (≥ 10 mutations/megabase), and (**d**) OS for high PD-L1 subset (≥ 50%).

Using the Kaplan–Meier method, median PFS was 3.7 months in patients with CDKN2A LOF tumors versus 7.1 months in those with CDKN2A wild-type tumors (Fig. 3a,*p* = 0.034). Similarly, median OS was 11.8 months versus 22.1 months (*p* = 0.043) for CDKN2A LOF and wild-type tumors, respectively (Fig. 3b). The negative impact on PFS (HR 2.15, 95% CI 1.22–3.75, *p* = 0.0091) and OS (HR 2.77, 95% CI 1.48–5.14, *p* = 0.0018) of CDKN2A LOF held on multivariate analysis (*full* model) including age, sex, BMI, ECOG, TMB, PD-L1 status, and ICB indication (Supplemental Tables 2 and 3, column 4). Using backwards selection criteria without pre-specified variables, the *final* model for PFS included sex, TMB, ICB indication and CDKN2A (HR 1.66, 95% CI 1.02–2.63, *p* = 0.041), whereas the *final* model for OS included ECOG, BMI, and CDKN2A (HR 2.08, 95% CI 1.21–3.49, *p* = 0.0087) (Supplemental Tables 2 and 3, column 5).

The impact of CDKN2A LOF was further examined in the high TMB and high PD-L1 patient subsets that typically experience improved ICB response. In patients with high TMB tumors (≥ 10 mutations/megabase), median OS was 10.5 months in the CDKN2A LOF group versus 22.3 months in the CDKN2A wild-type group (Fig. 3c, *p* = 0.069). In patients with tumors exhibiting PD-L1 ≥ 50%, median OS was 11.1 months versus 24.2 months by the same stratification (Fig. 3d, *p* = 0.020). By contrast, in patients with PD-L1 low tumors (< 50%), median OS was 17.0 months for CDKN2A WT and 13.9 months for CDKN2A LOF patients (*p* = 0.32), respectively. Similarly, in patients with TMB low tumors (< 10 mutations/megabase), median OS was 22 months for CDKN2A WT and 13.9 months for CDKN2A LOF patients (*p* = 0.31), respectively. On pre-specified multivariate analysis including CDKN2A mutation status, PD-L1 ≥ 50%, and TMB ≥ 10 mutations/megabase, CDKN2A status continued to be independently associated with PFS (HR 1.73, 95% CI 1.02–2.88, *p* = 0.042) and OS (HR 1.96, 95% CI 1.10–3.43, *p* = 0.0024). These findings suggest that the differences in OS observed between CDKN2A WT and LOF patients is in large part driven by the negative prognostic impact on patients with PD-L1 high or TMB high tumors.

Given the above findings, we examined the potential prognostic impact of CDKN2A in an independent external dataset from MSKCC that has been previously reported. Between 2011 and 2017, 240 patients with NSCLC underwent MSK-IMPACT genomic sequencing and were radiologically evaluable for treatment response following ICB. Given that ICB had not yet been approved for the off protocol treatment of NSCLC until 2015, many patients were heavily pre-treated and therefore those who had received > 5 prior lines of therapy were excluded to ensure a comparable cohort. Patients without available PD-L1 or TMB data were also excluded in order to allow for the control of known predictors of ICB response (final validation subset, N = 83). Overall survival was not publicly available. Patients with CDKN2A LOF tumors versus those with CDKN2A wild-type tumors exhibited an increased genome alteration fraction (mean: 37% vs. 16%, 2-tailed Student’s t-test *p* < 0.0001), but otherwise exhibited similar clinical and molecular features, including TMB and PD-L1 expression. Median PFS in the CDKN2A LOF versus wild-type groups was 2.1 months versus 4.8 months (*p* = 0.069), respectively (Supplemental Fig. 1). On pre-specified multivariate analysis including CDKN2A mutation status (LOF vs. wild-type), PD-L1 status (≥ 1% vs. 0%), and TMB (continuous variable) consistent with the reported MSKCC analysis, CDKN2A LOF continued to be independently associated with worsened PFS (HR 1.94, 95% CI 1.0–3.5, *p* = 0.048).

In addition, we used a permutation test for the multivariate Cox regression models (controlling for PD-L1 and TMB) to test the robustness of our PFS and OS findings. We randomized the labels of CDKN2A LOF and WT in our cohorts (keeping the number of LOF patients constant) 10,000 times to assess the likelihood that a *p* value at least as extreme as the *p* value reported in our analyses (p-prime) would be obtained by chance. For each trial, we computed the Wald *p* value (p-hat) for the CDKN2A LOF term of the Cox survival model given the permuted labels. We then calculated a “*p* value of *p* values” as the fraction of the 10,000 permutations for which p-hat ≤ p-prime, which represents the likelihood of obtaining a *p* value at least as significant as that observed in our survival model by chance. In the multivariate PFS and OS permutation analyses of the UCMC cohort, 5% and 3% of permuted *p* values were at least as extreme as the reported *p* values in our analyses. Similarly, in the multivariate PFS permutation analysis of the MSKCC validation cohort, 4% of permuted *p* values were at least as extreme as the reported *p* value. Taken together, these findings demonstrate that 5% or less of permuted *p* values are as extreme as the reported multivariate *p* values from the UCMC and MSKCC cohorts, which supports the conclusion that there is a small probability that our findings occurred by chance.

Finally, we sought to demonstrate the predictive specificity of CDKN2A LOF for clinical outcomes in the ICB setting rather than simply on the overall prognosis of NSCLC. We queried three independent external datasets of patients with NSCLC (N = 1913) who were treated with primarily surgery and cytotoxic therapies in the pre-immunotherapy era^3,26,27^. While CDKN2A LOF was associated with worsened OS on univariate Cox proportional hazards analysis in two of these cohorts (*data not shown*), this effect was abrogated when controlling for confounding variables. In particular, the frequency of CDKN2A LOF increased with more advanced tumor stage in all three cohorts (CDKN2A LOF incidence in early stage vs. advanced stage cohort 1 [N = 1144]: 28% vs. 35% *p* = 0.016; cohort 2 [N = 586]: 16% vs. 25% *p* = 0.015; cohort 3 [N = 183]: 12% vs. 28% *p* = 0.11). Multivariate Cox models including stage and CDKN2A LOF demonstrated no independent impact of CDKN2A status on patient outcomes in any of these cohorts (Supplemental Table 5).

### CDKN2A loss-of-function and response rate

**Figure 4:**
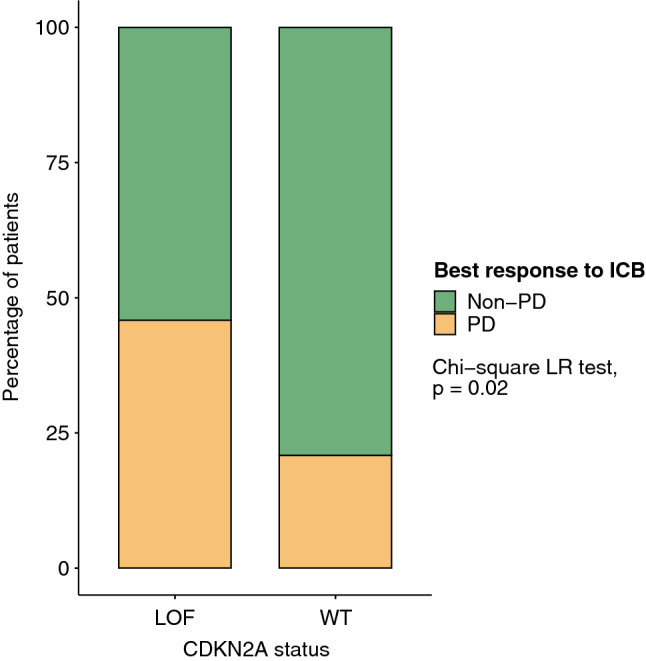
CDKN2A LOF and ICB response. Disease control rate (non-PD vs. PD) for CDKN2A wild-type (WT, N = 72) and loss-of-function (LOF, N = 24) tumors with evaluable lesions. Non-evaluable lesions could not be measured due to pleural effusions, interval lung collapse, and/or irregular lesion contours as per RECIST version 1.1 guidelines.

A total of 96 patients had lesions that were evaluable per RECIST version 1.1 criteria prior to ICB initiation. The breakdown of best ICB response was as follows: 1 (1%) with CR, 32 (34%) with PR, 36 (38%) with SD, and 26 (27%) with progressive disease (PD). The overall ORR was 35% and the DCR was 73%. Patients who experienced PD as best response had significantly worse PFS (*p* < 0.0001) and OS (*p* < 0.0001) as compared with those who experienced CR/PR or SD as best response (Supplemental Fig. 2a,b). We found that CDKN2A LOF tumors had significantly lower DCR and were twice as likely to exhibit disease progression when compared to CDKN2A wild-type tumors (PD as best response in 46% vs. 21%, Chi-squared *p* = 0.021, Fig. 4 and Supplemental Fig. 3); this result was most pronounced in those treated with ICB for metastatic disease (48% vs. 23%, Chi-squared *p* = 0.033). There was a numerically but not statistically lower ORR rate in CDKN2A LOF versus wild-type tumors (29% vs. 38%, *p* = 0.46).

We examined the impact of CDKN2A LOF on response rates to ICB monotherapy vs. ICB combination therapy. DCR was greater for ICB combination therapy vs. ICB monotherapy (80% vs. 66%, Chi-squared *p* = 0.11). In patients who received ICB monotherapy, DCR was 76% for CDKN2A WT versus 33% for CDKN2A LOF tumors (Chi-squared *p* = 0.007). By contrast, in patients who received ICB combination therapy, DCR was 82% for CDKN2A WT versus 75% for CDKN2A LOF tumors (Chi-squared *p* = 0.59). Taken together, these findings suggested that the negative prognostic impact of CDKN2A LOF is greatest for patients who received ICB monotherapy at least in part due to the reduced response rate to ICB monotherapy vs. combination therapy. Interestingly, ICB combination therapies may lead to increased response rates as compared to ICB monotherapy in patients with CDKN2A LOF.

## Discussion

In this study, we demonstrate that genomic alterations leading to LOF of CDKN2A are associated with poor clinical outcomes in a large cohort of patients with advanced NSCLC treated with ICB. In particular, CDKN2A LOF is associated with a two-fold increased rate of disease progression on anti-PD-1/PD-L1 therapy and a halving of median PFS and OS, including in the subsets predicted to respond most favorably to ICB (PD-L1 ≥ 50% or TMB ≥ 10 mutations/megabase). The deleterious effect of CDKN2A LOF remained when accounting for known predictive factors of ICB response. This finding was validated in an independent cohort of ICB treated patients but not in the context of three independent, non-ICB treated cohorts, suggesting a specific predictive role of CDKN2A LOF in the setting of ICB. This represents a novel and potentially targetable finding in NSCLC.

CDKN2A is an important tumor suppressor and one of the most frequently silenced genes in human malignancy^18^. In NSCLC, and specifically in lung adenocarcinoma, large genomic consortia have demonstrated silencing of CDKN2A through genetic or epigenetic mechanisms in up to 40% of tumors^3^. Furthermore, a growing body of evidence implicates CDKN2A LOF in tumor biology and outcome. Pre-clinical studies utilizing various models of NSCLC suggest that CDKN2A LOF independently contributes to an aggressive tumor phenotype^29,30^, whereas recent clinical studies in pancreatic adenocarcinoma^31^, melanoma^19^, and urothelial carcinoma^20^ demonstrate an association between CDKN2A alteration and worsened OS and ICB resistance. To our knowledge, the only published study that analyzed the role of CDKN2A in ICB resistance in NSCLC found a non-significant trend between CDKN2A LOF status and OS (WT vs. LOF, OS HR 1.3, *p* = 0.11). The data presented here is therefore the first to demonstrate a clear negative impact of CDKN2A LOF on oncologic outcomes in NSCLC patients treated with ICB.

Though the advent of ICB has revolutionized the treatment of NSCLC, ORRs remain limited for most patient cohorts^5,8^ and current predictive biomarkers have significant limitations in predicting clinical outcomes. Principal among these has been the lack of currently actionable therapeutic vulnerabilities implied by known genomic biomarkers, such as TMB and KRAS/STK11 co-mutation. In the setting of KRAS/STK11 co-mutation, this may change in the future with the recent FDA approval of the KRAS(G12C) inhibitor sotorasib^32^ and pre-clinical data suggesting that KRAS(G12C) inhibitors drive anti-tumor immunity^33^. CDKN2A LOF suggests another immediate and biologically plausible target.

CDKN2A encodes p14ARF and p16INK4a, two protein products that play pivotal roles in apoptosis and cell cycle regulation, respectively^18^. One of the primary mechanisms of action of anti-PD-1/PD-L1 therapy is the reversal of the T-cell exhausted state and a concomitant increase in cytotoxic T-cell mediated killing. This effector function is partially reliant upon remnant tumor cell apoptotic pathways of which p14ARF—through its actions as an MDM2 inhibitor—is a major component^34^. Unfortunately, early clinical results with small molecule MDM2 inhibitors have been disappointing and therefore preclude the use of these agents in reconstituting this aspect of CDKN2A function^35,36^.

On the other hand, p16INK4a regulates the cell cycle largely through inhibition of CDK4/6^37^. Unlike MDM2 and KRAS inhibitors, CDK4/6 inhibitors are widely used in the clinic based on several seminal phase III studies in metastatic breast cancer^38,39^. The phase II pragmatic basket TAPUR trial enrolled 29 heavily pre-treated patients with CDKN2A-altered NSCLC treated with palbociclib and demonstrated a DCR of 31%. Interestingly, the majority of patients who exhibited at least 16 weeks of durable control had received ICB as their immediately antecedent therapy^21^. Given that emerging data demonstrates that CDK4/6 inhibitors lead to both PD-L1 up-regulation and the activation of immune surveillance^40,41^, combinations of CDK4/6 inhibitors and ICB deserve study. Though several trials are investigating the combination of CDK4/6 inhibition and anti-PD-1/PD-L1 therapy in advanced breast cancer (NCT02778685, NCT04360941), such an approach has to our knowledge not been attempted in NSCLC.

There are several limitations to our findings. The first is the retrospective nature of our study. We strove to minimize bias by including all patients with advanced NSCLC who underwent OncoPlus testing as per institutional policy, by excluding patients with NGS performed after the initiation of ICB, and by conducting an external validation. Though the latter was only borderline significant on log-rank testing for PFS (*p* = 0.069), this was likely due to the small sample size of the MSKCC validation cohort (N = 83) and the presence of confounding variables (PD-L1, TMB). Indeed, on subsequent MVA controlling for these factors, CDKN2A LOF was significantly associated with PFS (*p* = 0.048). The second limitation is twofold: our analysis cannot define whether CDKN2A LOF is truly predictive of ICB response rather than simply clinically prognostic and that the existence of other immune-related genes on chromosome 9p may also contribute to our findings. However, the fact that outcomes were similarly poor in patients with CDKN2A mutation as in those with CDKN2A copy number loss suggests that LOF at the CDKN2A locus itself impacts PFS and OS in our ICB-treated cohort. A third limitation is the lack of data regarding CDKN2A promoter hypermethylation in our dataset. Interestingly, if CDKN2A promoter methylation has a similarly negative impact on outcome as CDKN2A gene loss/mutation, then the differences in ICB response and outcome between patients with CDKN2A-silenced tumors and those with true CDKN2A wild-type tumors are likely greater than those reported here, which warrants further investigation.

In conclusion, CDKN2A LOF negatively impacts clinical outcomes in advanced NSCLC treated with ICB, doubling rates of disease progression and halving PFS and OS, even in high PD-L1 and high TMB tumors. This novel finding should be further investigated and, if validated, presents a potential therapeutic target in a large subset of patients with NSCLC.

## Supplementary Information

Supplementary Information 1.

Supplementary Information 2.

## Data Availability

De-identified genomic and clinical data are presented in Supplemental Table 4.
