# Supplementary material for: CDKN2A loss-of-function predicts immunotherapy resistance in non-small cell lung cancer
Source: Sci Rep. 2021 Oct 8;11:20059. doi: 10.1038/s41598-021-99524-1 (PMC8501138; doi:10.1038/s41598-021-99524-1)
Supplement: Supplementary file 1 — Supplementary Information 1. [file 41598_2021_99524_MOESM1_ESM.pdf]

**Supplemental Methods and Materials:**

**OncoPlus:** Targeted next-generation sequencing (OncoPlus) of 1,212 genes was performed at the University of Chicago (UCMC) Clinical Genomic and Molecular Pathology Laboratory between May 2016 and June 2019 as previously described[22]. Briefly, sequencing reads with a mapping quality greater than zero were initially aligned to the human genome (hg19) using Burrows-Wheeler Alignment version 0.7.2[41]. Realignment was performed using ABRA version 0.96[42] to improve the detection of complex genomic variants. Additionally, duplicate reads produced during the PCR amplification process were removed using the Picard MarkDuplicates version 1.92 or 2.81 (http://broadinstitute.github.io/picard/; Broad Institute). Variants were called using samtools mpileup version 0.1.19 and known OncoPlus artifacts were excluded. Variants in a set of 154 clinically-relevant cancer-related genes that were clinically validated were retained for further analysis **(Supplemental Table 6)**. Variants were annotated using Alamut Interactive BioSoftware version 1.4.4 or 1.11 to obtain known population allele frequencies from 1000 Genomes, gnomAD, and ESP, the predicted functional consequence of each mutation, and SIFT algorithm prediction of effects on protein function. Variants with a variant allele fraction (VAF) of >5% were filtered out if present in one of the three population databases at a frequency of >1%. Because no matched normal tissue was sequenced, all remaining variants with a VAF >5% were further subject to manual expert molecular pathology review to identify putative artifacts, benign germline variants, and determine variant pathogenicity using an internal database of previously reported variants[43]. Novel variants were manually reviewed and their putative etiology and pathogenicity determined by a molecular pathologist. Variants were interpreted based on the 2017 AMP, ASCO, and CAP standards and guidelines for the interpretation and reporting of sequence variants in cancer[23].

**Copy Number Variation Analysis:** Copy number variant calling was performed on all samples undergoing OncoPlus testing. CNVKit version 8.8.2 was used to detect whole gene copy

number variations through a clinically validated process[44]. Briefly, following removal of PCR duplicates (as described above), BAM files were analyzed with CNVKit using a historical pool of non-malignant samples as a baseline, which were established during the original validation of the assay. Samples sequenced after April 2018 were additionally normalized to the pooled control sample on each flow cell and a set of seven previously sequenced controls to minimize capture-to-capture variability. 143 clinically relevant, clinically validated, cancer-related genes were analyzed for copy number alterations **(Supplemental Table 6)**. Genes were called as having equivocal copy-number loss if 50% of bins across the gene had a fold-change (FC) of <0.6 and the gene was on the laboratory's clinical list of tumor suppressor genes. Full gene loss required average FC of bins across the gene of <0.5, with all 143 genes eligible for calling. Genes were called as having equivocal copy-number gain if the average FC of gene bins was 2-4, while full amplification required average FC >4. Similar to equivocal losses, equivocal gain was reportable per the test if the gene is on the laboratory's list of key oncogenes and if manual pathologist review determined that the gain was gene-specific (reportable) vs. the result of a whole chromosome or regional gain (considered clinically non-reportable). Full amplification is reportable for all 143 genes without any specific requirement for manual triage.

**Tumor Mutational Burden Quantification:** A subset of genes from the full OncoPlus panel of 1,212 genes was used to calculate the tumor mutational burden (TMB) of each tumor sample. The subset of 1,135 genes used in the TMB module do not include regions determined to be problematic due to significant sequence homology, low sequencing depth, and/or repetitive regions. TMB was defined as the number of coding mutations per megabase of genome sequenced [total bait size sequenced was 2.8 megabases]. Variants were excluded if they had a Phred quality score ≤30, sequencing depth of less than 50x, or VAF <10%. Furthermore, variants were filtered out if they were present in 1000 Genomes or ExAC databases using the most recent version at the time of annotation performed by Alamut. However, variants filtered by

the population databases were rescued if they had >10 entries in COSMIC and an ExAC frequency of < 0.001, or if the variant was an indel, present in the ExAC database with QC flags (RF or AC0), and had an ExAC frequency of no greater than 1%.

**Supplemental Figure 1. CDKN2A LOF and survival in MSKCC cohort.** Kaplan-Meier curves of PFS in CDKN2A wild-type (WT) versus CDKN2A loss-of-function (LOF) tumors for MSKCC validation cohort.

**Supplemental Figure 2. Association of ICB response and survival.** Kaplan-Meier curves of **(A)** PFS and **(B)** OS based on best RECIST version 1.1 response to ICB. CR/PR, complete response/partial response; SD, stable disease; PD, progression of disease.

**Supplemental Figure 3. Spider Plots by CDKN2A Status.** Spider plots of ICB responses in patients with measurable lesions: **(A)** CDKN2A WT (N = 61) and **(B)** CDKN2A LOF tumors (N = 16).

**Supplemental Table 1: Treatment Details**

| | N (%) |
|---|---|
| **ICB Agent*** | |
| Pembrolizumab | 80 (58%) |
| Nivolumab | 19 (14%) |
| Nivolumab/Ipilimumab | 18 (13%) |
| Atezolizumab | 17 (12%) |
| Durvalumab | 11 (8%) |
| **ICB Combination** | |
| ICB Alone | 89 (64%) |
| Chemo + ICB | 39 (28%) |
| Chemo-ICB + Targeted Therapy^ | 9 (6%) |
| ICB + Targeted Therapy^ | 2 (1%) |
| **Chemotherapy Class** | |
| Platinum-Containing | 53 (98%) |
| Other | 1 (2%) |
| **Prior Lines of Systemic Therapy** | |
| 0 | 94 (68%) |
| 1 | 35 (25%) |
| ≥2 | 10 (7%) |
| **Radiotherapy** | |
| Yes | 104 (75%) |
| No | 35 (25%) |

*Includes all ICB received during the entirety of each patient’s treatment course, with several patients receiving multiple ICB agents. ^All ICB + targeted therapy patients treated on clinical trials

**Supplemental Table 2: Analysis of Variable Association with PFS**

| | UVA HR (95% CI) | UVA P Value | Full MVA P value | Final MVA P value |
|---|---|---|---|---|
| **ENTIRE COHORT** | | | | |
| Age (continuous) | 1.01 (0.99-1.03) | 0.48 | 0.20 | - |
| Sex (M vs. F) | 1.45 (0.95-2.20) | 0.085 | 0.17 | 0.11 |
| BMI (continuous) | 0.98 (0.93-1.03) | 0.41 | 0.050 | - |
| ECOG (0-1 vs. 2-3) | 0.90 (0.50-1.79) | 0.75 | 0.55 | - |
| TMB | | | | |
| Continuous | 0.98 (0.96-1.00) | 0.080 | 0.092 | 0.073 |
| ≥ 10 vs. <10 | 0.84 (0.55-1.28) | 0.42 | - | - |
| ≥ 13.8 vs. <13.8 | 0.80 (0.50-1.25) | 0.33 | - | - |
| PD-L1 ≥ 50% vs. < 50% | 0.78 (0.47-1.25) | 0.30 | 0.47 | - |
| ICB Indication (Stage III vs. IV) | 0.52 (0.23-1.01) | 0.055 | **0.014** | **0.024** |
| CDKN2A (LOF vs. WT) | 1.64 (1.02-2.59) | **0.043** | **0.0091** | **0.041** |

Values bolded are significant at threshold of Chi-squared $p < 0.05$ using Cox proportional hazards analysis. All continuous variable hazard ratios (HRs) are per unit change in regressor.

**Supplemental Table 3: Analysis of Variable Association with OS**

| | UVA HR (95% CI) | UVA P Value | Full MVA P value | Final MVA P Value |
|---|---|---|---|---|
| **ENTIRE COHORT** | | | | |
| Age (continuous) | 1.02 (0.99-1.04) | 0.17 | **0.016** | - |
| Sex (M vs. F) | 1.25 (0.78-2.00) | 0.36 | 0.55 | - |
| BMI (continuous) | 0.94 (0.88-0.99) | **0.024** | **0.0017** | **0.0074** |
| ECOG (0-1 vs. 2-3) | 0.49 (0.28-0.95) | **0.034** | **0.034** | **0.017** |
| TMB | | | | |
| Continuous | 0.99 (0.96-1.01) | 0.35 | 0.57 | - |
| ≥ 10 vs. <10 | 1.20 (0.75-1.92) | 0.45 | - | - |
| ≥ 13.8 vs. <13.8 | 0.88 (0.51-1.46) | 0.63 | - | - |
| PD-L1 ≥ 50% vs. < 50% | 1.07 (0.62-1.83) | 0.82 | 0.86 | - |
| ICB Indication (Stage III vs. IV) | 0.52 (0.18-1.17) | 0.12 | **0.020** | - |
| CDKN2A (LOF vs. WT) | 1.84 (1.06-3.06) | **0.053** | **0.0018** | **0.0087** |

Values bolded are significant at threshold of Chi-squared $p < 0.05$ using Cox proportional hazards analysis. All continuous variable hazard ratios (HRs) are per unit change in regressor.

**Supplemental Table 4: De-identified Clinical and Genomic Data from UCMC Cohort**

**Supplemental Table 5: Multivariate Analysis of Stage and CDKN2A Alteration on Clinical Outcomes in non-ICB Treated External Cohorts**

| | **Variable** | **MVA HR (endpoint)** | **Lower 95%** | **Upper 95%** | **P-value** |
|---|---|---|---|---|---|
| **Cohort 1 (Campbell et al.)** | | | | | |
| | Stage (3 vs. 1) | 2.12 (OS) | 1.562 | 2.860 | **<.0001** |
| | Stage (4 vs. 1) | 2.37 (OS) | 1.296 | 4.001 | **0.007** |
| | CDKN2A (LOF vs. WT) | 1.23 (OS) | 0.954 | 1.574 | 0.110 |
| **Cohort 2 (TCGA)** | | | | | |
| | Stage (3 vs. 1) | 3.48 (OS) | 2.363 | 5.087 | **<.0001** |
| | Stage (4 vs. 1) | 3.80 (OS) | 2.116 | 6.428 | **<.0001** |
| | CDKN2A (LOF vs. WT) | 1.28 (OS) | 0.903 | 1.776 | 0.163 |
| **Cohort 3 (Imielinski et al.)** | | | | | |
| | Stage (3 vs. 1) | 2.28 (PFS) | 1.233 | 3.946 | **0.010** |
| | Stage (4 vs. 1) | 0.59 (PFS) | 0.204 | 1.325 | 0.218 |
| | CDKN2A (LOF vs. WT) | 1.16 (PFS) | 0.693 | 1.853 | 0.555 |

Values bolded are significant at threshold of Chi-squared $p < 0.05$ using multivariate Cox proportional hazards analysis (MVA).

**Supplemental Table 6: List of Analyzed Genes**

**Genes analyzed for mutations and insertions/deletions (154 genes):** ABL1, AKT1, ALK, APC, ARID1A, ARID2, ASXL1, ATM, ATR, ATRX, AXL, B2M, BAP1, BCOR, BCORL1, BIRC3, BLM, BRAF, BRCA1, BRCA2, BTK, CALR, CBL, CBLB, CCND1, CCND2, CCND3, CDH1, CDK4, CDK6, CDKN2A, CEBPA, CHEK1, CHEK2, CSF1R, CSF3R, CTCF, CTNNA1, CTNNB1, CUX1, CXCR4, DAXX, DDR2, DDX3X, DDX41, DICER1, DNMT3A, EGFR, EP300, EPHA3, EPHA5, ERBB2, ERBB3, ERBB4, ERCC3, ESR1, ETV6, EZH2, FANCA, FAT3, FBXW7, FGFR1, FGFR2, FGFR3, FH, FLT3, FOXL2, GATA1, GATA2, GNA11, GNAQ, GNAS, GRIN2A, H3F3A, HIST1H3B, HIST1H3C, HNF1A, HRAS, IDH1, IDH2, IKZF1, ITPKB, JAK2, KDM6A, KDR, KIT, KMT2A, KRAS, MAP2K1, MAPK1, MDM2, MET, MLH1, MLH3, MPL, MRE11A, MSH2, MSH6, MTOR, MYC, MYCN, MYD88, NBN, NF1, NF2, NFE2L2, NOTCH1, NOTCH2, NPM1, NRAS, PALB2, PBRM1, PDGFRA, PDGFRB, PHF6, PIK3CA, PIK3CB, PIK3R1, PLCG2, POLE, POT1, PPP2R1A, PTCH1, PTEN, PTPN11, RAD21, RAD51, RB1, RET, RUNX1, SDHB, SDHC, SDHD, SETBP1, SF3B1, SMAD4, SMARCB1, SMC1A, SMC3, SMO, SRSF2, STAG2, STAT3, STAT5B, STK11, TERT (promoter only), TET2, TP53, TSC1, TSC2, U2AF1, VHL, WT1, ZRSR2

**Genes analyzed for copy number variations (143 genes):** ABL1, AKT1, ALK, APC, ARID1A, ARID2, ASXL1, ATM, ATR, AXL, B2M, BAP1, BIRC3, BLM, BRAF, BRCA1, BRCA2, CALR, CBL, CBLB, CCND1, CCND2, CCND3, CDH1, CDK4, CDK6, CDKN2A, CEBPA, CHEK1, CHEK2, CSF1R, CSF3R, CTCF, CTNNA1, CTNNB1, CUX1, CXCR4, DAXX, DDR2, DDX41, DICER1, DNMT3A, EGFR, EP300, EPHA3, EPHA5, ERBB2, ERBB3, ERBB4, ERCC3, ESR1, ETV6, EZH2, FANCA, FAT3, FBXW7, FGFR1, FGFR2, FGFR3, FH, FLT3, FOXL2, GATA2, GNA11, GNAQ, GNAS, GRIN2A, H3F3A, HIST1H3B, HIST1H3C, HNF1A, HRAS, IDH1, IDH2, IKZF1, ITPKB, JAK2, KDR, KIT, KMT2A, KRAS, MAP2K1, MAPK1, MDM2, MET, MLH1, MLH3, MPL, MRE11A, MSH2, MSH6, MTOR, MYC, MYCN, MYD88, NBN, NF1, NF2, NFE2L2, NOTCH1, NOTCH2, NPM1, NRAS, PALB2, PBRM1, PDGFRA, PDGFRB, PIK3CA, PIK3CB, PIK3R1, PLCG2, POLE, POT1, PPP2R1A, PTCH1, PTEN, PTPN11, RAD21, RAD51, RB1, RET, RUNX1, SDHB, SDHC, SDHD, SETBP1, SF3B1, SMAD4, SMARCB1, SMC3, SMO, SRSF2, STAT3, STAT5B, STK11, TERT, TET2, TP53, TSC1, TSC2, U2AF1, VHL, WT1

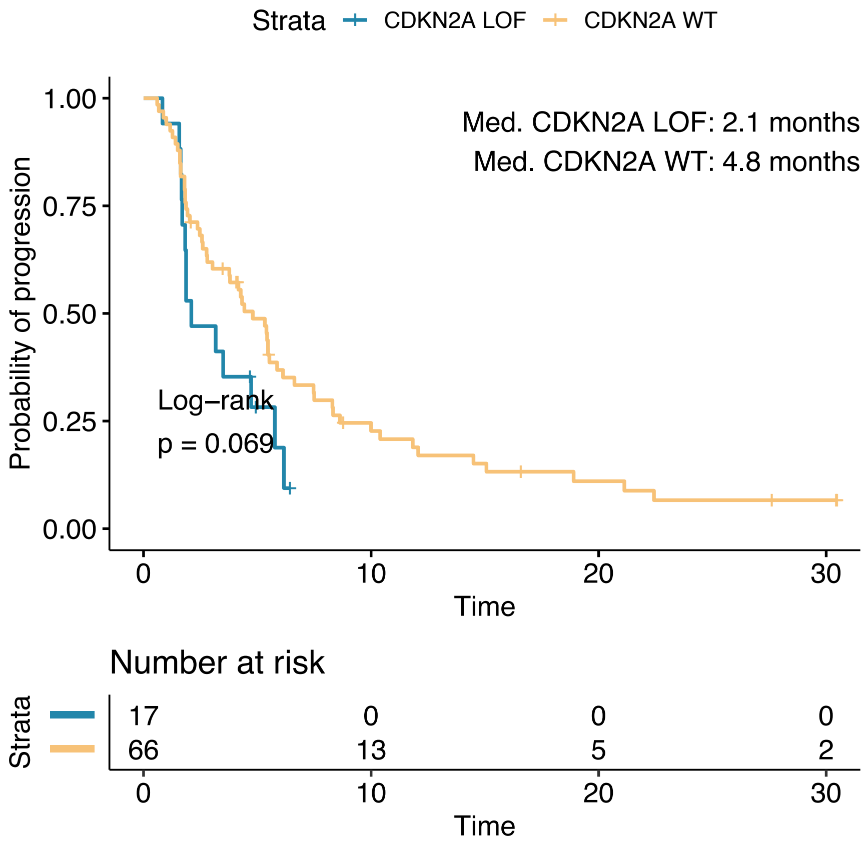


**Supplementary Figure 1**

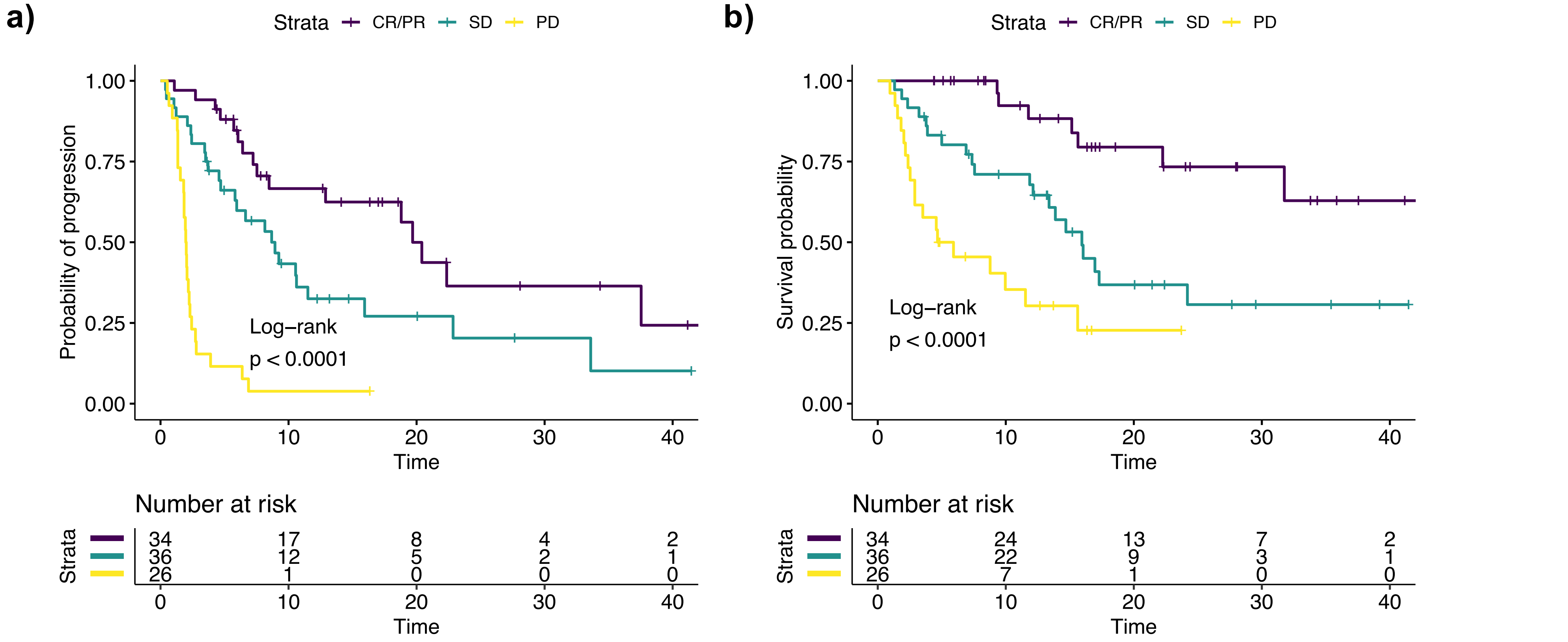


**Supplementary Figure 2**

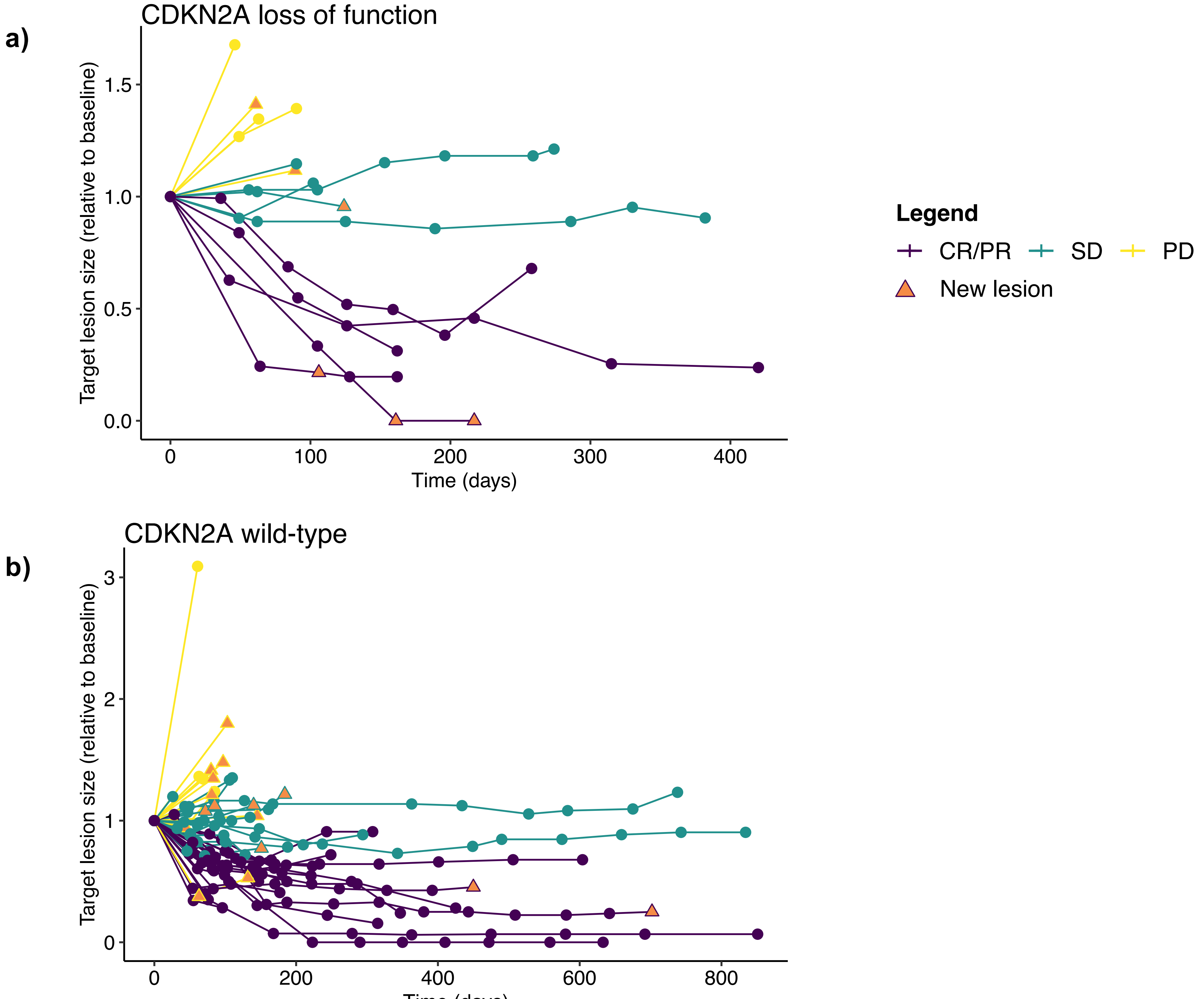


**Supplementary Figure 3**
